# rCCLasso: a robust framework for microbial correlation network analysis reveals age-related microbial dynamics

**DOI:** 10.3389/fcimb.2026.1828471

**Published:** 2026-08-17

**Authors:** Tianyi Xie, Jie Zhou, Yue Wang

**Affiliations:** 1 Department of Statistics, Oregon State University, Corvallis, OR, United States; 2 School of Mathematical Sciences, Capital Normal University, Beijing, China; 3 Department of Biostatistics and Informatics, University of Colorado Anschutz Medical Campus, Aurora, CO, United States

**Keywords:** Cauchy combination test, compositional data, hub taxa, network density, robust inference

## Abstract

**Introduction:**

The human gut microbiome continues to evolve beyond early adulthood, yet most microbiome aging studies focus on changes in individual taxa or overall diversity, leaving microbial interaction dynamics largely unexplored. Correlation-based microbial networks offer an interpretable framework for studying such interactions but are challenging to estimate from compositional microbiome data. Although compositionality-aware methods such as CCLasso provide principled multivariate inference, we identify a previously overlooked limitation: sensitivity to random seeds, which leads to unstable correlation estimates and irreproducible significance assessments.

**Methods:**

To address this issue, we propose Robust CCLasso (rCCLasso), a statistically rigorous framework that stabilizes microbial correlation estimation by integrating CCLasso outputs across multiple runs. rCCLasso aggregates sparse correlation estimates using median-based integration with positive-definite projection and combines run-specific inference through the Cauchy combination test with an additional stability criterion to control type-I error. The method is naturally parallelizable and computationally scalable.

**Results:**

Simulation studies demonstrate that rCCLasso improves inferential stability, type-I error control, and power relative to the original CCLasso. Applying rCCLasso to data from over 4,000 healthy adults in the American Gut Project (ages 18--101), we uncover age-related microbial network dynamics, characterized by marked fluctuations from early to mid-adulthood, followed by a relatively stable phase and a substantial decline in network strength in the elderly group.

**Discussion:**

Together, these results establish rCCLasso as a robust and interpretable framework for studying microbial networks in aging research.

## 1 Introduction

It is increasingly known that the human gut microbiome undergoes dynamic changes throughout life. In early life, microbial communities fluctuate rapidly, shaped by factors such as delivery mode, breastfeeding, diet, and antibiotic exposure (Derrien et al., 2019; Tanaka and Nakayama, 2017). During infancy, specific taxa such as Bifidobacterium and Bacteroides dominate, while other bacterial groups, including members of the Firmicutes and Bacteroidetes phyla, become more prominent as the microbiome matures (Yatsunenko et al., 2012). Measures of microbial richness and evenness, such as alpha-diversity, increase substantially during this period (Xu et al., 2019). Although adulthood is generally characterized by relative microbial stability, emerging evidence indicates that the gut microbiome continues to evolve well beyond early adulthood. With age, microbial composition often undergoes gradual shifts, including decreases in overall diversity (Salazar et al., 2017; Wilmanski et al., 2021), reductions in beneficial taxa such as Faecalibacterium prausnitzii, and increases in potentially harmful groups like Proteobacteria (Claesson et al., 2012; Jeffery et al., 2016; Ghosh et al., 2020). Interestingly, several studies report that older individuals, including centenarians, maintain or even exhibit higher levels of alpha-diversity compared to younger adults (Odamaki et al., 2016; Biagi et al., 2016; Salazar et al., 2017; Wang et al., 2019), suggesting that the gut microbiome may continue to diversify in later life. These findings highlight a more complex relationship between aging and gut microbial dynamics throughout the entire lifespan.

Most studies examining the microbiome-aging link have focused on changes in the abundance of individual taxa or on broad measures of diversity, without taking a systems-biology perspective. Consequently, little is known about how microbial interactions evolve throughout adult life. Understanding these dynamics is critical because the gut microbiome functions as a complex, interconnected ecosystem that regulates immune responses, metabolizes nutrients, and maintains gut barrier integrity (Wiertsema et al., 2021; Takiishi et al., 2017; Zheng et al., 2020). Shifts in microbial interactions may influence inflammation (Zheng et al., 2020) and metabolic balance (Cox et al., 2022; Olofsson and Bäckhed, 2022), with potential implications for predicting health outcomes and guiding interventions. Studying the dynamics of microbial networks therefore offers a complementary perspective for understanding the relationship between the gut microbiome and healthy aging.

Correlation-based approaches represent one of the most widely used and interpretable strategies for constructing microbial networks. A fundamental challenge for these approaches arises from the compositional nature of microbiome data. Microbiome measurements are typically expressed as relative abundances constrained to sum to one, which can induce spurious correlations even when the underlying absolute abundances are independent (Gloor et al., 2017; Carr et al., 2019). Consequently, the naïve application of standard correlation measures, such as Pearson or Spearman correlations, can lead to distorted network structures and misleading biological interpretations (Friedman and Alm, 2012; Lovell et al., 2015; Kurtz et al., 2015). To address this issue, several compositionality-aware methods have been proposed for microbial correlation estimation. For example, SparCC (Friedman and Alm, 2012) estimates correlations between latent absolute abundances using log-ratio transformations under a sparsity assumption, and has been shown to perform well in heterogeneous compositional settings (Weiss et al., 2016). However, an important limitation of SparCC is that its pairwise estimation procedure does not guarantee that the inferred covariance matrix is positive definite, and the resulting correlation coefficients may even fall outside the valid range of [−1,1] (Fang et al., 2015). These issues complicate interpretation and can invalidate downstream analyses, such as clustering or dimension reduction, which rely on positive semidefinite correlation or covariance matrices. A more recent method, CCLasso (Fang et al., 2015), estimates microbial correlations using a d-trace loss function with an *ℓ*_1_ penalty. Unlike SparCC, which relies on pairwise estimation, CCLasso adopts a multivariate framework and ensures that the estimated correlation matrix is positive definite. The core estimation procedure in CCLasso is implemented via an efficient alternating direction method of multipliers (ADMM; Zhang and Zou, 2014) based on an augmented Lagrangian formulation, enabling scalable computation for high-dimensional data. The same authors later proposed a computationally faster variant, fastCCLasso (Zhang et al., 2024), that accelerates optimization. Although the original CCLasso publications focused on point estimation without formal inference, the publicly available R package includes a de-biasing procedure to produce entry-wise *p*-values. These *p*-values provide a measure of uncertainty and facilitate downstream inference procedures such as false discovery rate control and robust edge selection in microbial networks.

Despite CCLasso’s rigor and effectiveness for estimating microbial correlations, we identify a practical limitation of this approach that has been largely overlooked. Specifically, the CCLasso estimator exhibits mild to moderate numerical instability across repeated runs, even when applied to the same dataset with the same tuning parameters. To the best of our knowledge, this instability originates from the stochastic bootstrap de-biased inference procedure used by CCLasso for statistical inference. We provide a detailed discussion of this mechanism in the following section. Although these differences are often modest in magnitude, they may affect the identification of significant correlations and pose challenges for reproducibility and interpretation. To address this limitation, we develop a *Robust CCLasso* (rCCLasso) pipeline that stabilizes correlation estimation and inference by aggregating results across repeated algorithm runs under different random seeds using statistically rigorous procedures. In each run, we obtain a CCLasso sparse correlation matrix estimate and corresponding entry-wise *p*-values. To aggregate correlation estimates across runs, we compute the median estimate for each matrix entry and subsequently project the resulting matrix onto the space of positive definite matrices if necessary. For statistical inference, we combine entry-wise *p*-values across runs using the Cauchy combination test (Liu and Xie, 2020), which remains valid under arbitrary dependence and is well-suited to correlated run-specific estimates. To mitigate the potential sensitivity of the Cauchy test to a small number of extremely small *p*-values, we further impose a stability criterion requiring that at least *α* × 100% of runs yield nominal significance at level *α*, thereby improving robustness. Because all runs are independent, the pipeline is naturally parallelizable, making the computational cost manageable even for large numbers of repetitions. Through extensive simulations, we demonstrate that the proposed approach improves reproducibility while achieving better control of type-I error and increased power compared to the original CCLasso method.

To investigate how microbial interactions evolve across adulthood, we applied the proposed rCCLasso to a large-scale dataset from the American Gut Project (McDonald et al., 2018), comprising over 4,000 healthy U.S. adults aged 18 to 101 years. Participants were divided into seven age groups with approximately balanced sample sizes. Using rCCLasso, we observed noticeable age-dependent changes in microbial network densities, including substantial fluctuations during early adulthood (18–37) and a significant decline between the 58–64 and 65+ groups. From a taxon-level perspective, Bacilli and Coriobacteriia emerged as hub taxa starting in the 31–37 age group and remained central through the elderly group, suggesting some consistency in key network components across the adult lifespan. Nevertheless, less than 10% of microbial edges were consistently detected across all age groups, while roughly 50% of edges were either never observed or appeared in only a single age group, highlighting substantial variability in network structure with age. Overall, these findings demonstrate the utility of rCCLasso for revealing biologically meaningful and previously unobserved patterns in age-related microbial network dynamics, and underscore its potential as a robust and interpretable tool for studying microbial interactions.

## 2 Results

### 2.1 Simulations

**Figure 1:**
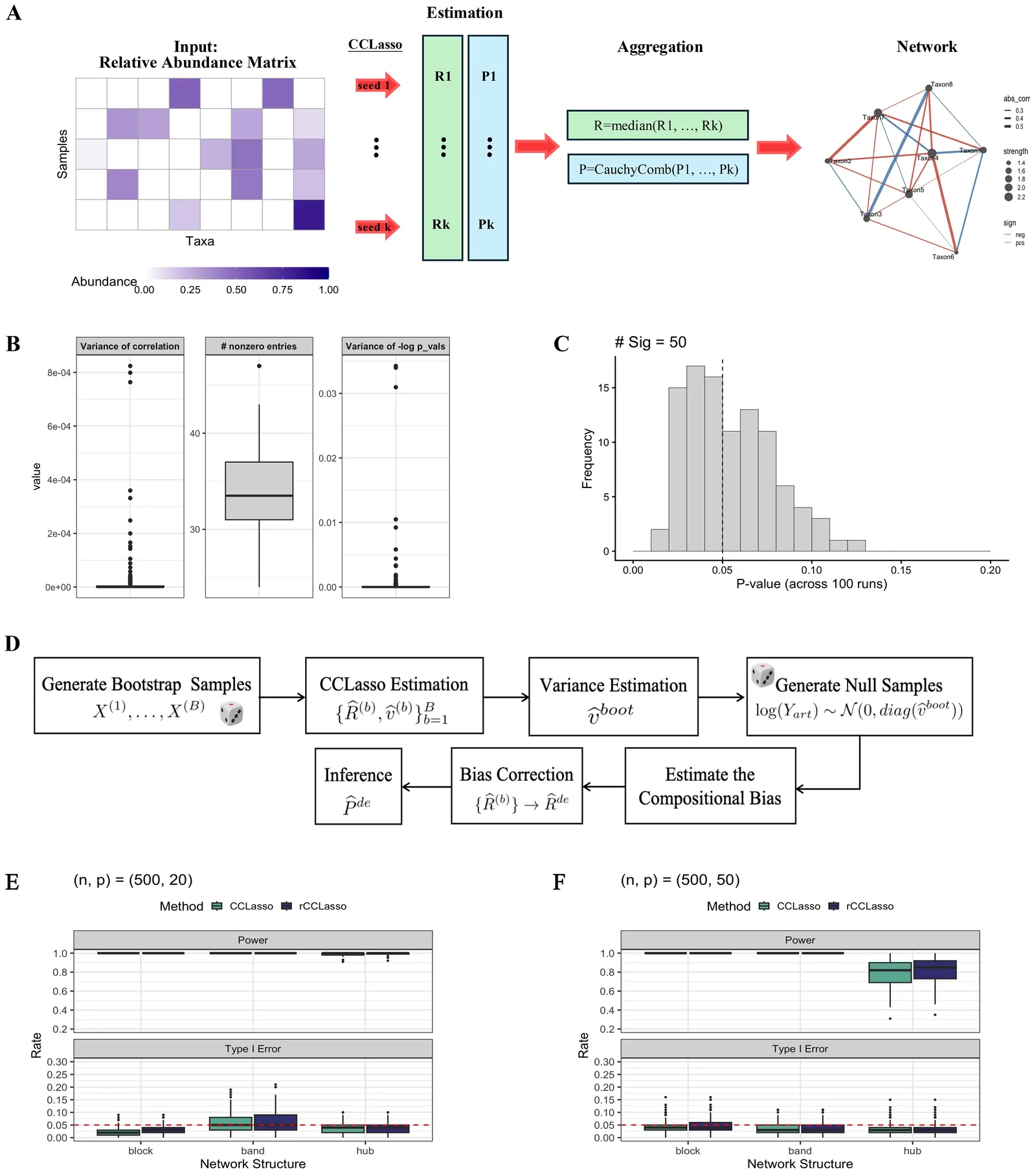
Overview of the rCCLasso framework and simulation results. **(A)** Schematic of the rCCLasso workflow. Starting from a sample-by-taxa relative abundance matrix, CCLasso is applied repeatedly with different random seeds. rCCLasso aggregates correlation estimates via the entry-wise median and combines *p*-values using the Cauchy combination test. Significant associations are determined after FDR and stability control and visualized as a weighted network, with node size representing centrality, edge width reflecting correlation strength, and edge color indicating positive (red) or negative (blue) correlations. **(B)** Simulation using one data set with a fixed tuning parameter (*λ* = 0.4) across 100 CCLasso runs. Boxplots of entry-wise correlation estimates and *p*-values show mild variation, while the number of non-zero correlations exhibits substantial variability, highlighting instability in network inference. **(C)** Simulation using the same data as in B with the optimal tuning parameter (*λ* = 0.103) across 100 runs. Entry-wise *p*-values vary little in B, but significance for a selected entry fluctuates: roughly 50 runs are significant (*p <* 0.05) and 50 are not, illustrating instability in inference. **(D)** Workflow of the bootstrap de-biased inference procedure in CCLasso. Dice icons denote stochastic sampling steps. **(E, F)** Entry-wise empirical power and Type I error of CCLasso and rCCLasso under two simulation settings: (*n,p*) = (500,20) **(E)** and (*n,p*) = (500,50) **(F)**. Three network structures (block, band, and hub) were considered. rCCLasso shows type-I error closer to 0.05 and comparable or higher power than CCLasso across all structures.

We provide an overview of rCCLasso in **Figure 1A**. rCCLasso is a robust extension of CCLasso (Fang et al., 2015) designed to address the lack of numerical stability in the original method. Although CCLasso offers multivariate estimation and positive-definite covariance guarantees, we identify that its correlation estimates and associated *p*-values can vary across independent runs, raising concerns about reproducibility and interpretability.

To systematically evaluate this issue, we conducted a simulation study to demonstrate that the numerical instability in CCLasso can lead to unstable and potentially misleading inference, with significance assessments varying markedly across repeated runs. In the second simulation study, we show that rCCLasso not only substantially improves inferential stability but also achieves better control of type-I error while maintaining or enhancing statistical power relative to the original CCLasso. Detailed simulation designs are included in Section 3.2, and below, we discuss the primary findings.

**Figure 1B** quantifies the numerical instability of CCLasso under repeated 100 runs on the same simulated dataset with a fixed tuning parameter (*λ* = 0.4). First, for each matrix entry, we computed the sample variance of the 100 correlation estimates; across entries, these variances range from 0 to 8.24 × 10^−4^, with a median of 5.95 × 10^−8^, suggesting that entry-wise variability is modest in magnitude. However, this seemingly mild variation can translate into substantial differences at the network level. Across the 100 runs, the number of non-zero off-diagonal correlation estimates ranges from 24 to 47 (median 33.5). Because non-zero entries are typically interpreted as edges in a microbial network, such instability can lead to markedly different network structures across repeated runs, raising concerns about reproducibility and interpretability. We also examined the variance of entry-wise −log_10_-transformed *p*-values, which range from 0 to 0.0343 across entries, with a median of 7.08×10^−7^, again appearing small overall. Nevertheless, such small fluctuations may substantially affect inference. To demonstrate this, using the same simulated dataset with another fixed tuning parameter (*λ* = 0.103, the optimal value selected by CCLasso), for the (5,16) entry of the correlation matrix (**Figure 1C**), 50% of runs yield a *p*-value below 0.05 while the remaining 50% exceed 0.05, rendering the significance decision fairly arbitrary across runs.

The numerical instability observed in CCLasso appears to arise from the stochastic bootstrap-based de-biased inference procedure used for statistical inference. Although this procedure is implemented in the original CCLasso software, it is not explicitly described in the original publication. We therefore investigated its implementation in detail. As illustrated in **Figure 1D**, the inference procedure contains two stochastic sampling steps: bootstrap resampling and artificial null sample generation. Both steps may contribute to variability across different random seeds. A detailed description of this procedure is provided in the Methods section and **Supplementary Section S1**. Although this variability could, in principle, be eliminated by fixing the random seed for both sampling steps, doing so would make the resulting inference dependent on a single arbitrary seed, with no justification for selecting one seed over another. This observation motivates the proposed rCCLasso framework. Rather than relying on a single random realization, rCCLasso aggregates information across multiple independent realizations to reduce seed-dependent variability and improve the robustness of both estimation and statistical inference.

We further conducted comprehensive simulation studies to compare CCLasso and rCCLasso in terms of type-I error control and statistical power. To better reflect the characteristics of microbiome sequencing data, we considered two simulation schemes: the logistic-normal (LN) model and the logistic-normal–multinomial (LNM) model (Xia et al., 2013; Tian et al., 2023). The latter further incorporates sequencing depth (library size) variation. The results under the LN model are presented in the **Figure 1E, F**, whereas the corresponding LNM results are provided in **Supplementary Section S2**. In each setting, three correlation structures were considered: block, band, and hub. For each zero entry, the type-I error was calculated as the proportion of rejected null hypotheses across 100 simulated data sets, while power was computed as the rejection proportion across simulated data sets for non-zero entries. Across both dimensional settings and all correlation structures, rCCLasso achieves type-I error rates that are overall closer to the nominal level (i.e., 0.05) compared to the original CCLasso, indicating improved calibration. At the same time, rCCLasso maintains comparable or higher power, with particularly noticeable gains under the hub structure. These results demonstrate that aggregation across repeated runs not only enhances stability but also improves detection accuracy. Similar conclusions were obtained under the LNM model, which explicitly accounts for sequencing depth variation, indicating that the performance of rCCLasso is robust to the underlying data-generating mechanism (**Supplementary Section S2**). In addition, because the number of repeated runs (*B*) is the primary tuning parameter of rCCLasso, we conducted a sensitivity analysis under the same LN simulation settings using different values of *B*. As shown in **Supplementary Section S3**, the proposed method exhibits stable performance across a range of *B* values.

### 2.2 The AGP data

**Figure 2:**
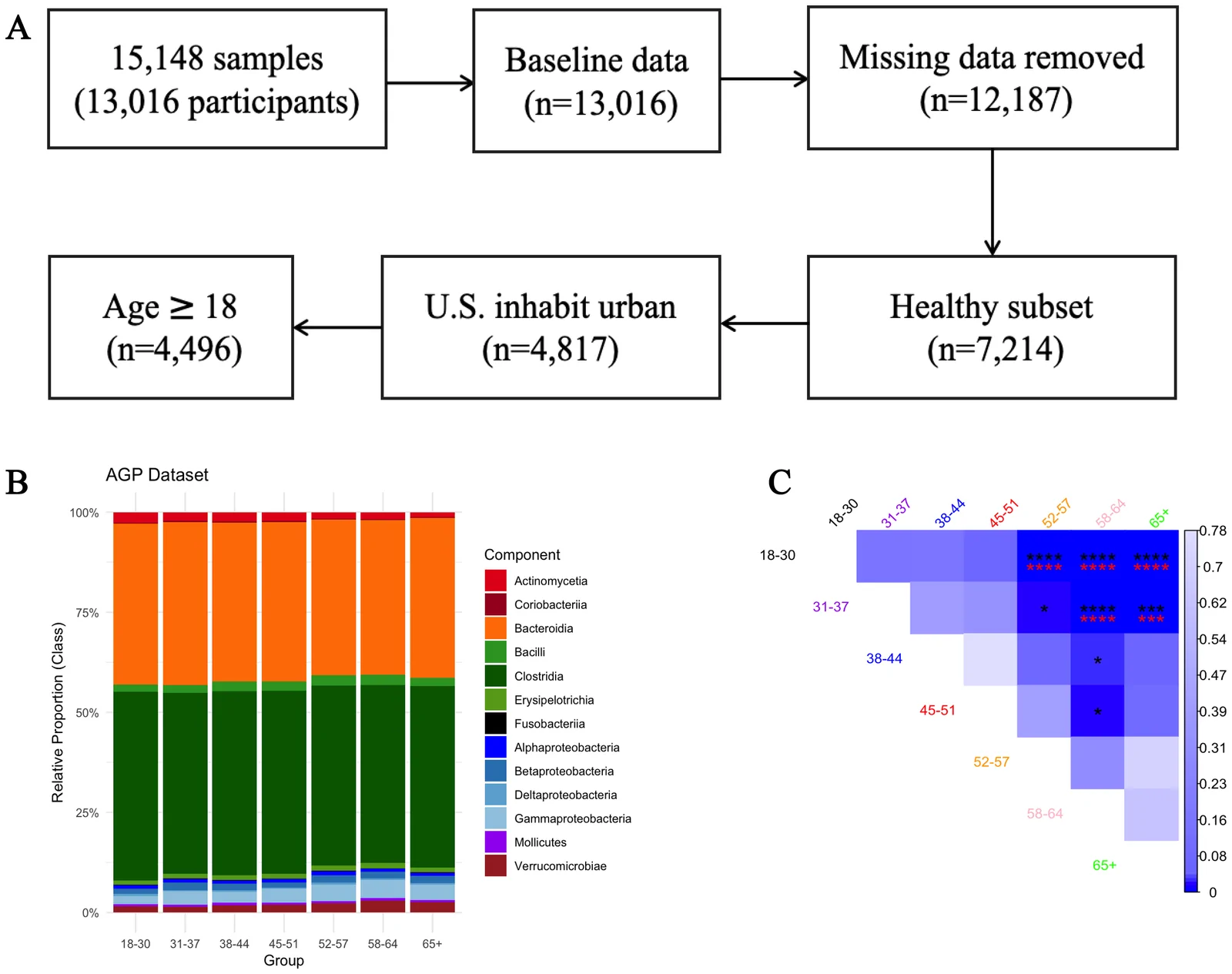
Sample processing and relative abundance across age groups. **(A)** Sample inclusion pipeline. The dataset initially contains 15,148 samples from 13,016 participants. The first measurement from each participant was selected as the baseline sample, and individuals with missing age information were excluded. The analysis was further restricted to a healthy subset comprising participants with no self-reported history of inflammatory bowel disease (IBD), diabetes, or antibiotic use in the past year. Only participants residing in the United States and aged 18 years or older were retained. **(B)** Mean relative abundance of microbial taxa at the class level across seven age groups. **(C)** Heatmap of pairwise comparisons of class-level relative abundances between age groups based on the James test. Significance levels are denoted as ∗(*p* ≤ 0.05), ∗∗(*p* ≤ 0.01), ∗∗∗(*p* ≤ 0.001), and ∗∗∗∗(*p* ≤ 0.0001). Black asterisks indicate nominal *p*-values, whereas red asterisks indicate FDR-adjusted *p*-values.

We analyzed microbiome data from the American Gut Project (AGP), a large-scale cohort comprising 15,148 samples from 13,016 participants. To investigate microbial patterns associated with healthy aging after adulthood, we restricted our analysis to healthy U.S. adults, yielding 4,496 fecal samples from unique individuals aged 18 to 101 years. A sample exclusion diagram is provided in **Figure 2A**. To characterize age-related differences in the gut microbiome, participants aged ≥ 65 years were defined as the elderly group, while those aged 18–64 years were partitioned into six groups, each containing approximately 600 samples to ensure balanced comparisons. The genus-level microbiome data exhibited substantial zero inflation, with a median zero proportion of 99.97% across taxa (IQR: 99.67% – 100%). To mitigate the impact of excess zeros, we conducted the primary analysis at the class taxonomic level. This aggregation reduced sparsity, improved the stability of network estimation and inference, and also reduced the computational burden of repeated model fitting. In addition, class-level networks provide a more interpretable summary of broad microbial interaction patterns and age-related ecological shifts. Two classes, *Chloroplast* and *4C0d-2*, were further removed because they are not considered resident human gut taxa. Both belong to the phylum *Cyanobacteria*: *Chloroplast* sequences are typically derived from dietary plant DNA and are commonly treated as contaminants in gut microbiome studies (Hanshew et al., 2013), while *4C0d-2* has shown inconsistent taxonomic placement across databases and studies (Hemmings et al., 2017). Additional details on sample processing are provided in Section 3.3.

Although it is not our primary focus, we begin by examining age-related changes in taxonomic relative abundance. The barplot in **Figure 2B** illustrates the class-level relative abundances of gut microbiota across different age groups. Aging is associated with a decreasing trend in *Actinobacteria* and increasing trends in *Verrucomicrobiae* and *Gammaproteobacteria*. Specifically, *Actinobacteria* exhibit a notably higher relative abundance in younger individuals, approximately twice that observed in older adults (2.6% at ages 18–30 vs. 1.2% at ages 65+). In contrast, the relative abundances of *Verrucomicrobiae* and *Gammaproteobacteria* display a different pattern, nearly doubling with age up to 65, followed by a slight decline thereafter. The *Proteobacteria* phylum (including *Alpha-*, *Beta-*, *Delta-*, and *Gammaproteobacteria*) shows an increase in abundance after age 52 compared to earlier age groups (see blue regions in bar plot: 4.7% at ages 18–30 vs. 7.4% at ages 52–57), primarily driven by changes in *Gammaproteobacteria*. In contrast, other classes within *Proteobacteria*, such as *Alphaproteobacteria*, remain relatively stable across both younger and older age groups. The relative abundance of *Bacteroidia* exhibits a downward trend between ages 18 and 64, followed by an upward trend. *Clostridia*, a dominant class-level taxon within the *Firmicutes* phylum, decreases from 46.9% in the younger group (18–30) to 44.8% in the 31–37 age group, and then stabilizes at this level in the elderly group (65+). Among other classes within *Firmicutes*, *Bacilli* show a slightly higher relative abundance during mid-adulthood (38–64), with comparable levels observed in both younger and older groups. In contrast, the abundance of *Erysipelotrichi* remains relatively stable across all age groups. *Coriobacteriia* also exhibits a consistently low and stable relative abundance (around 0.3%). *Fusobacteriia* is the least abundant class across all age groups, reaching only 0.04% in the 58–64 age group.

The heatmap in **Figure 2C** summarizes pairwise comparisons of relative abundances across age groups. The results indicate that overall gut microbial composition changes substantially with age; however, these changes do not follow a simple linear trajectory. Compared with the younger groups (18–30 and 31–37), pronounced differences first emerge in the 52–57 age group, characterized by a distinct shift in microbial composition that persists into older ages. This apparent “jump” suggests that middle age (52–57) may represent a critical transition point in gut microbiota dynamics, separating early adulthood from later life stages.

**Figure 3:**
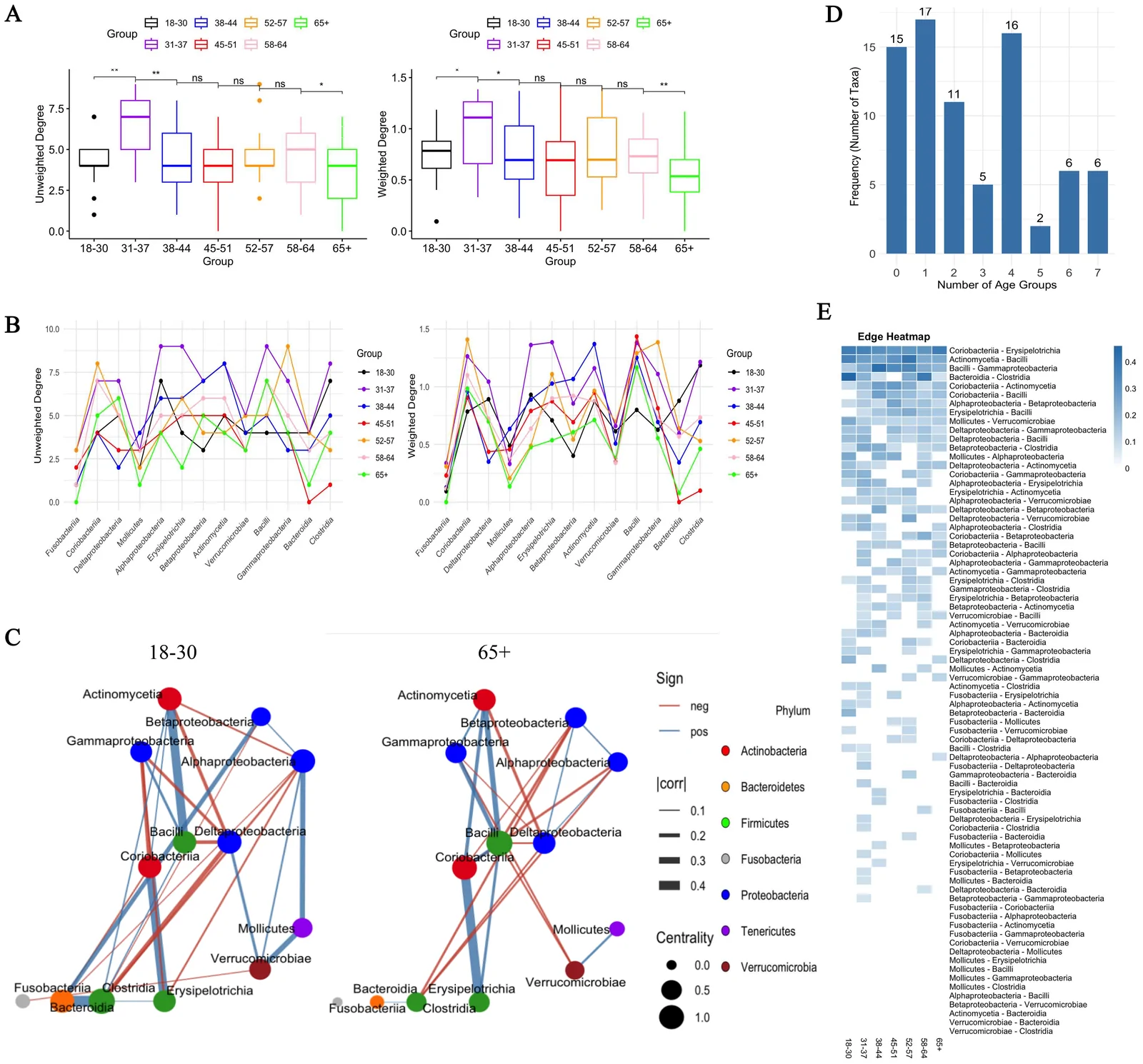
Microbial network analysis of AGP data. **(A)** Boxplots of unweighted (left) and weighted (right) node degrees across age groups. Paired Wilcoxon signed-rank tests were used to assess differences between consecutive groups. Significant changes are observed from 18–30 to 31–37, 31–37 to 38–44, and 58–64 to 65 +. **(B)** Line plots of node degree for each microbial class across age groups (left: unweighted; right: weighted). **(C)** Age-group-specific networks. Only the 18–30 and 65+ age groups are shown for visualization; the remaining age-group networks are presented in Supplementary Section S5. Nodes are colored by phylum and sized by node degree. Edges represent pairwise correlations, with red indicating positive and blue indicating negative correlations. Edge width reflects the absolute correlation strength. **(D)** Barplot summarizing the number of microbial pairs detected as significant in *k* age-group-specific networks (*k* = 0,1*,…*,7): 6 pairs detected in all age groups, while 15 detected in none of the age groups. **(E)** Heatmap of microbial correlation strengths for each of the 78 microbial pairs across all seven age groups. Significance levels are denoted as *(p ≤ 0.05), **(p ≤ 0.01), ***(p ≤ 0.001), and ****(p ≤ 0.0001).

We next applied the proposed rCCLasso to construct a microbiome network for each age group, with edge widths proportional to the estimated robust microbial correlations. For each network, we computed both the unweighted node degree and the weighted node degree for every taxon and assessed their differences between adjacent age groups. As shown in **Figure 3A**, both measures reveal a similar pattern across age groups. The overall node degree is relatively low in the 18–30 group, followed by a significant increase in the 31–37 group. This increase is then followed by a significant decline in the 38–44 group, returning to a level comparable to that observed in the youngest group. These results suggest notable fluctuations in microbial connectivity during early adulthood. After age 38, node degrees remain relatively stable across subsequent age groups up to 58–64. Finally, a significant decline is observed in the elderly group (65+), indicating a reduction in overall microbial network connectivity at older ages.

To further characterize node-specific connectivity and identify hub taxa, **Figure 3B** displays the node degree of each microbial class across age groups. From the unweighted analysis, the hub structure of the microbial network varies considerably across age groups, with only limited overlap in the taxa occupying central network positions. *Bacilli* emerges as one of the most persistent hubs, but only appears in three age groups (31–37, 58–64, and 65+), while *Coriobacteria* also functions as a hub in mid-to-late adulthood (52–57 and 58–64). In contrast, several hubs appear to be age-group specific. For example, *Clostridia* serves as the primary hub in the youngest group (18–30), *Mollicutes* and *Erysipelotrichia* are prominent in the 31–37 group, and *Actinomycetia* becomes central in the 38–44 group. In the 52–57 group, *Coriobacteriia* and *Gammaproteobacteria* emerge as hubs, whereas *Bacilli* and *Deltaproteobacteria* dominate the network structure in the 65+ group. Notably, the 45–51 age group does not exhibit a clearly dominant hub, suggesting a more evenly distributed connectivity structure during this stage. When edge weights (correlation strengths) are incorporated, the hub structure becomes more consistent across age groups. From the weighted analysis, both *Bacilli* and *Coriobacteriia* function as a hub from the 31–37 group onward and remain central through the elderly group (65+). These findings suggest that accounting for correlation strength reduces some of the apparent variability in hub identity and highlights a smaller set of taxa that consistently exert strong interaction influence across age groups.

Finally, we visualize the age-group-specific microbial correlation networks in **Figure 3C**, which provide a detailed view of the correlation structures inferred by rCCLasso across different age groups. To further summarize the consistency of microbial associations across age groups, we examined each pair of microbes (78 pairs in total) and counted the number of age groups in which the pair was identified as a significant edge. As shown in **Figure 3D**, only 6 pairs exhibit significant correlations across all age groups, whereas 32 pairs (approximately 50%) appear in at most one age group, including 15 pairs that are not detected in any group. From **Figure 3E**, which visualizes the strength of microbial correlations for each pair across all age groups, we observe that those six pairs consistently detected in every age group are primarily associated with the hub taxa identified from the weighted degree analysis, namely *Bacilli* and *Coriobacteriia*. The only exception is the connection between *Alphaproteobacteria* and *Betaproteobacteria*, which shows a consistent but relatively weak correlation across all age groups. This pattern may reflect coordinated variation between these two classes within the same phylum, *Proteobacteria*, which can arise from shared ecological responses rather than direct microbial interactions. Beyond the consistently detected pairs, several microbial associations exhibit clear age-specific patterns. For example, the correlation between *Deltaproteobacteria* and *Clostridia* is detected only in the youngest (18–30) and oldest (65+) groups, suggesting a potential re-emergence of this association later in life. The pair *Betaproteobacteria–Bacteroidia* shows a strong correlation exclusively in the youngest group. In addition, correlations involving *Alphaproteobacteria* and *Clostridia* with *Actinomycetia* are observed in the younger groups (before age 37) but disappear in subsequent age groups. This suggests that these connections may weaken or reorganize as the gut microbiome matures across adulthood.

## 3 Methods

### 3.1 rCCLasso: model, algorithm, and applications

**Notation**. We consider modeling the relative abundances of p taxa measured on n subjects. Let $x_{ij}$ denote the observed relative abundance of taxon j for subject i, where i=1,…,n and j=1,…,p. By definition, $x_{ij}\geq 0$ and $\sum_{j=1}^{p}x_{ij}=1\text{ for each }i.$ The observed data are collected in a matrix $X\in {\mathbb{R}}^{n\times p}$. The relative abundances are linked with underlying latent absolute abundances (counts) $y_{ij}\geq 0$ through


$$x_{ij}=\frac{y_{ij}}{\sum_{j=1}^{p}y_{ij}}.$$

Our objective is to infer the covariance structure of the latent log-abundances. Specifically, we aim to estimate $\text{Cov }(\text{log }y_{ij},\text{log }y_{ik})$ for any pair (j,k) and test whether it equals zero. The logarithmic transformation is applied to biological count data to stabilize variance and better approximate normality.

A brief review of CCLasso. Let ${\text{Σ}}_{\text{log }y}$ and ${\text{Σ}}_{\text{log }x}$ denote the covariance matrices of the log-transformed latent absolute abundances ( $y_{ij}$) and the log-transformed observed relative abundances ( $x_{ij}$), respectively. CCLasso (Fang et al., 2015) estimates a sparse, positive-definite ${\text{Σ}}_{\text{log }y}$ by exploiting the following relationship between ${\text{Σ}}_{\text{log }y}$ and ${\text{Σ}}_{\text{log }x}$:

(1) $${\text{Σ}}_{\text{log }x}={\text{Σ}}_{\text{log }y}-a1_{p}^{⊤}-1_{p}a^{⊤},$$

where $1_{p}\in {\mathbb{R}}^{p}$ is a p-dimensional vector of ones, and $a\in {\mathbb{R}}^{p}$ has entries $a_{j}=\text{Cov }(\text{log }y_{ij},\text{log }(\sum_{k=1}^{p}y_{ik}))-\text{Var }(\text{log }(\sum_{k=1}^{p}y_{ik}))/2$. Note that ${\text{Σ}}_{\text{log }x}$ can be directly estimated from the observed compositional data, whereas both ${\text{Σ}}_{\text{log }y}$ and a are unknown and must be estimated.

To eliminate the nuisance parameter **a** from (Equation 1), CCLasso introduces a matrix *F* satisfying rank(*F*) = *p* − 1 and *F***1***_p_*= 0, i.e., *F* is column-centered. Left- and right-multiplying (Equation 1) by *F* yields


$$F{\text{Σ}}_{\text{log }x}F^{⊤}=F{\text{Σ}}_{\text{log }y}F^{⊤},$$

where the nuisance term vanishes due to the centering property of *F*. Replacing Σ_log_*_x_*with its empirical estimate ${\hat{\text{Σ}}}_{\text{log }x}$ leads to the estimating equation


$$F{\hat{\text{Σ}}}_{\text{log }x}F^{⊤}=F{\text{Σ}}_{\text{log }y}F^{⊤}.$$

Under a sparsity assumption on Σ_log_*_y_*, CCLasso estimates Σ_log_*_y_*by solving the penalized optimization problem:


$${\hat{\text{Σ}}}_{\text{log }y}=\text{arg }\underset{\text{Σ}≻0}{\min}\{||F({\hat{\text{Σ}}}_{\text{log }x}-\text{Σ})F^{⊤}{||}^{2}+\lambda ||{\text{Σ}}^{-}{||}_{1}\},$$

where ||·|| denotes a matrix norm, $||{\text{Σ}}^{-}{||}_{1}=\sum_{i\neq j}|\sigma_{ij}|$ penalizes the off-diagonal entries to encourage sparsity, and λ>0 is a tuning parameter. An efficient alternating direction algorithm is proposed in Fang et al. (2015) to solve this optimization problem, with cross-validation used for tuning parameter selection. The original CCLasso framework focuses on parameter estimation and does not provide formal inference for individual entries. To facilitate hypothesis testing, the authors implemented a de-biasing procedure in their R package CCLasso, enabling entry-wise *p*-values for testing *H*_0_: Cov(log*y_ij_*,log*y_ik_*) = 0. We refer readers to the supplementary material of fastCCLasso (Zhang et al., 2024) for numerical evaluations of this hypothesis testing approach.

**CCLasso’s instability**. The estimator ${\hat{\text{Σ}}}_{\text{log }y}$ produced by CCLasso suffers from numerical instability even when the input data and the tuning parameter are held fixed. To the best of our knowledge, this instability stems from the stochastic bootstrap de-biased inference procedure for statistical inference. A complete algorithmic description is provided in **Supplementary Section S1**. Briefly, CCLasso first performs bootstrap resampling to obtain multiple correlation estimates and an empirical variance estimator. Based on the estimated variances, it then generates artificial null samples, estimates the systematic compositional bias under the null model, and constructs debiased correlation estimates together with the corresponding Student’s t-statistics and two-sided p-values(**Figure 2D**). From a methodological perspective, this inference procedure serves two purposes: the bootstrap stage stabilizes variance estimation in high-dimensional settings, whereas the debiasing stage corrects the shrinkage bias induced by the *l*_1_-type penalty, following the debiasing framework of Van de Geer et al. (2014) and Zhang and Zhang (2014). At the same time, the two stochastic sampling steps are embedded in this inference procedure: bootstrap resampling and artificial null sample generation. Both steps inevitably introduce variability across different random seeds, thereby contributing to the empirical instability observed in CCLasso. While generally small, these differences can create challenges for reproducibility and the interpretation of significant correlations (**Figure 2C**). One possible way to reduce this variability is to fix a random seed before running the CCLasso algorithm; however, there is no principled way to select an optimal seed, motivating the development of rCCLasso to stabilize estimates through aggregation across repeated algorithm runs.

**The rCCLasso algorithm**. rCCLasso (**Algorithm 1**) stabilizes CCLasso by aggregating results across repeated algorithm runs under different random seeds, combining correlations via median-based positive-definite projection, integrating p-values using the Cauchy combination test, and determining significance through FDR control together with a stability criterion across runs.

We perform B independent runs of CCLasso on the same data matrix $X\in {\mathbb{R}}^{n\times p}$, each initialized with a different random seed. Let $R^{\left(b\right)}\in {\mathbb{R}}^{p\times p}$ and $P^{\left(b\right)}\in {\mathbb{R}}^{p\times p}$ denote the correlation matrix and entry-wise p-value matrix obtained from the b-th run, for b=1,…,B. Each run selects its tuning parameter $\lambda^{\left(b\right)}$ via cross-validation. Although we previously showed that variability still arises even when the tuning parameter is fixed, here, we allow $\lambda^{\left(b\right)}$ to be chosen independently in each run to reflect practical data analysis, where cross-validation is used to optimize estimation performance.

After *B* runs, we obtain correlation estimates ${\left\{R^{\left(b\right)}\right\}}_{b=1}^{B}$. For each pair (i,j), we aggregate the run-specific estimates via the entry-wise median:


$${\tilde{R}}_{ij}=\text{median}\{R_{ij}^{\left(1\right)},\ldots ,R_{ij}^{\left(B\right)}\}.$$

This yields an aggregated matrix $\tilde{R}\in {\mathbb{R}}^{p\times p}$. We adopt the median, rather than the mean, to enhance robustness against occasional run-specific numerical instability, as the median is less sensitive to extreme realizations. Because the median is computed entry-wise, $\tilde{R}$ is not theoretically guaranteed to remain positive definite, even though each $R^{\left(b\right)}$ is positive definite. In practice, the cross-run variability is typically mild, and $\tilde{R}$ is positive definite in most realizations. When necessary, we enforce positive definiteness via projection:


$$\hat{R}=\text{arg }\underset{R≻0}{\min}||R-\tilde{R}{||}_{F}^{2},$$

where $||\cdot {||}_{F}$ denotes the Frobenius norm. This step minimally perturbs $\tilde{R}$ while ensuring that $\hat{R}$ is a valid correlation matrix.

For each pair (i,j), we combine the run-specific p-values $\left\{P_{ij}^{\left(1\right)},\ldots ,P_{ij}^{\left(B\right)}\right\}$ using the Cauchy combination test:


$$T_{ij}=\frac{1}{B}\sum_{b=1}^{B}\text{ tan}[(\frac{1}{2}-P_{ij}^{\left(b\right)})\pi ],\;{\tilde{P}}_{ij}=\frac{1}{2}-\frac{1}{\pi }\text{arctan }(T_{ij}).$$

Under the null hypothesis, ${\tilde{P}}_{ij}$ is approximately uniform, and validity holds under arbitrary dependence among ${\left\{P_{ij}^{\left(b\right)}\right\}}_{b=1}^{B}$ (Liu and Xie, 2020), which is appropriate since run-specific estimates are correlated. Because inference is performed over p(p−1)/2 taxa pairs, we apply a false discovery rate (FDR) controlling procedure (e.g., the Benjamini–Hochberg method) to ${\left\{{\tilde{P}}_{ij}\right\}}_{i<j}$, yielding adjusted p-values ${\tilde{P}}_{ij}^{\text{adj}}$ (Benjamini and Hochberg, 1995).

The Cauchy test tends to emphasize extremely small *p*-values, which improves power under sparse alternatives but may amplify isolated run-specific artifacts. To guard against such instability, we introduce an additional stability criterion. Define


$$m_{ij}=\sum_{b=1}^{B}1\{P_{ij}^{\left(b\right)}<\alpha \}.$$

Under the global null hypothesis, $E[m_{ij}]=\alpha B$, so *αB* represents the expected number of nominally significant outcomes by chance. We retain an edge (*i,j*) only if


$${\tilde{P}}_{ij}^{\text{adj}}<\alpha \;\text{and }m_{ij}>\alpha B,$$

ensuring that discoveries are supported both by global error control and by cross-run stability.

**Downstream network analyses**. We visualize the rCCLasso output as an undirected microbial association network, where each node represents a taxon and edges correspond to statistically significant correlations. Edge color encodes the direction of correlation (e.g., red for positive correlations and blue for negative correlations), while edge width is proportional to the estimated correlation magnitude, providing a visual representation of interaction strength. Beyond visualization, network structure can be summarized at two levels. For a single inferred network, standard graph-theoretic measures characterize both global and local structure. At the node level, we report both unweighted degree, i.e., the number of nonzero edges connected to a node, and weighted degree, defined as the sum of the absolute values of the robust correlation estimates connected to that node. At the network level, overall edge density quantifies connectivity and community organization.

Algorithm 1

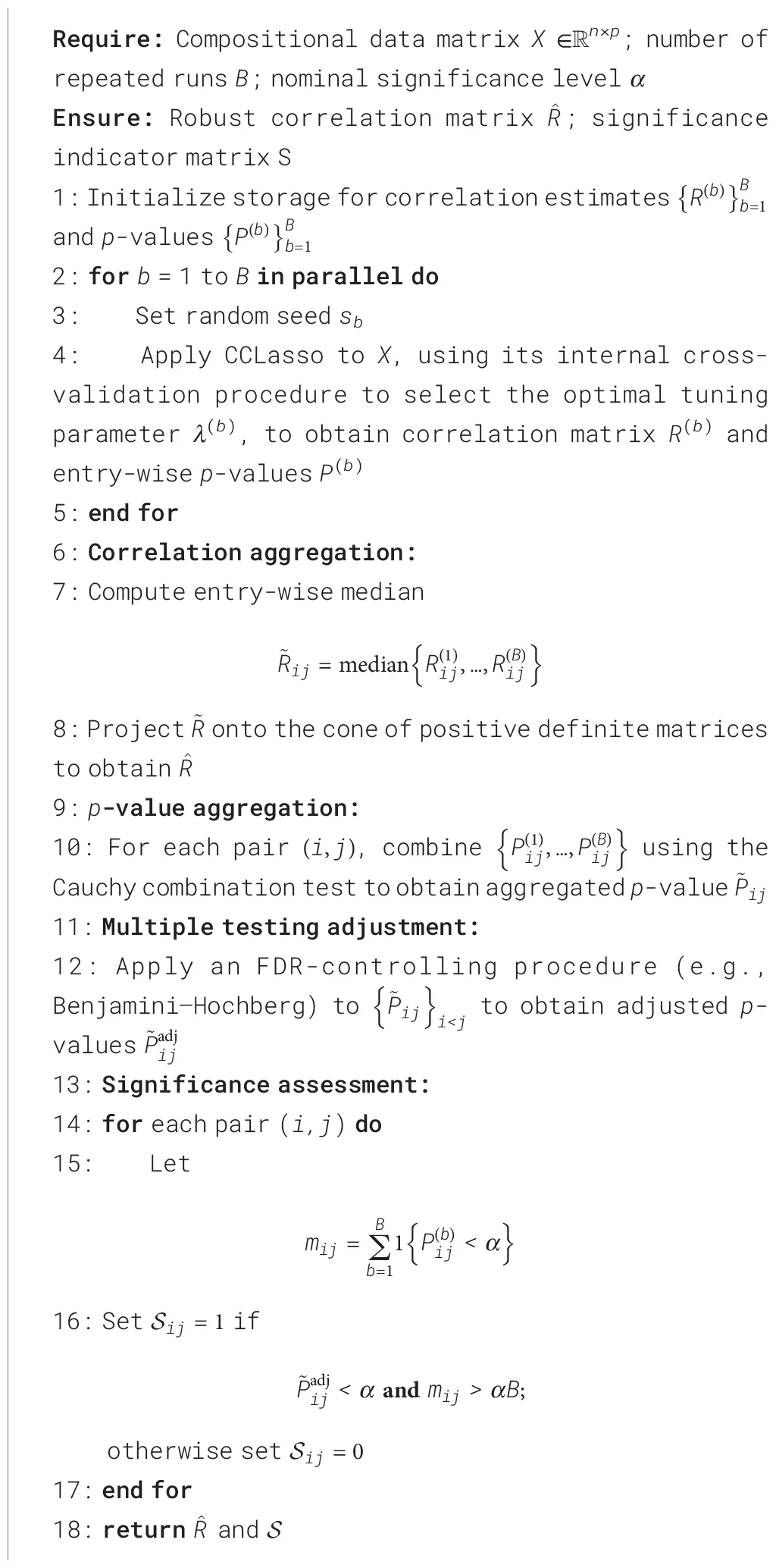

### 3.2 Simulation studies

**Compositional Data Generation**. The compositional data {**x**_1_*,…*,**x***_n_*} are generated from an additive logistic multivariate normal distribution LN(µ,Σ). In detail, we first generate **z** from a multivariate normal distribution *N*(*µ*,Σ) and map them to the simplex using the softmax transformation:


$$x_{i}=\frac{\exp(z_{i})}{\sum_{k=1}^{p}\exp(z_{k})}.$$

The elements of the mean vector *µ* were independently drawn from a uniform distribution *U*[−1,1].

**Simulation for assessing the variation of CCLasso**. To evaluate the numerical instability of CCLasso under varying random seeds, we simulated a single compositional dataset with *n* = 100 subjects and *p* = 20 nodes, assuming a random network structure. Specifically, each off-diagonal entry of the covariance matrix Σ was assigned a nonzero correlation with probability 0.3. The magnitudes of nonzero entries were set to ±1 with equal probability to represent both positive and negative edges, and the diagonal entries were adjusted to ensure that Σ was positive definite. We then converted Σ to a correlation matrix *R* and generated compositional data from a multivariate log-normal distribution, *LN*(*µ,R*). To isolate the effect of the tuning parameter, we fixed *λ* = 0.4 and ran the CCLasso algorithm 100 times using different random seeds. For each run, we recorded the estimated correlation matrix, the corresponding *p*-values, and the number of identified non-zero off-diagonal edges (**Figure 1B**).

To further investigate the impact of *p*-value variability on significance assessment, we repeated the analysis using the same simulated dataset with the CCLasso optimal tuning parameter (*λ* = 0.103) obtained via the golden-section search. Using the same 100 random seeds, we recorded the entry-wise *p*-values for each correlation. Notably, some entries exhibited substantial instability across runs, as illustrated by entry (5,16) in **Figure 1C**.

**Simulation studies comparing CCLasso and rCCLasso**. We extended the previous simulations to compare type-I error and power between CCLasso and rCCLasso. We considered *p* = 20 and 50 with *n* = 500 subjects. For each (*n,p*) combination, we simulated three realistic network structures following Fang et al. (2015):

1. Band network: Nodes *i* and *j* are connected if |*i* − *j*|≤ 3. Edge strengths are 0.6, 0.4, and 0.3 for |*i* − *j*| = 1,2,3, respectively.
2. Hub network: Two hub nodes for *p* = 20 and three for *p* = 50 are connected to all other nodes with probability 0.8. Edge strengths are drawn uniformly from [−0.8,−0.5] ∪ [0.5,0.8] for *p* = 20 and [−0.6,−0.4] ∪ [0.4,0.6] for *p* = 50. Non-hub nodes are connected only through hubs.
3. Block network: Nodes are partitioned into five equal-sized blocks. Within-block edges are present with probability 0.8 and strength 0.8, while between-block edges are absent (0).

For each network, the diagonal entries were adjusted to ensure positive definiteness, and the resulting covariance matrix was converted to a correlation matrix *R*. We generated 100 datasets from *LN*(*µ,R*) for each scenario and estimated correlation networks using CCLasso and rCCLasso. Type-I error was calculated as the proportion of true null edges (*R_ij_*= 0) incorrectly identified, and power was calculated as the proportion of true nonzero edges (*R_ij_*|= 0) correctly detected. Results are summarized in **Figure 1D** and E.

### 3.3 The AGP data analysis: sample preparation, data processing, and statistical comparisons

**AGP sample preparation**. The American Gut Project (AGP) was founded in November 2012 at the University of California San Diego as a large-scale, crowdsourced citizen science initiative. The primary objective of the AGP was to characterize the diversity and composition of the human gut microbiome across a broad population-level cohort, and to elucidate associations between microbial community structure and host factors including diet, lifestyle, and disease status (McDonald et al., 2018). The study employed a participatory design in which volunteers received a self-collection kit to obtain fecal samples, which were returned via mail for processing. Participants also completed a detailed questionnaire capturing general health history, disease status, dietary habits, and lifestyle variables.

The AGP dataset used in the present study comprises microbial sequence data from 15,148 samples collected across 13,016 human participants. After excluding participants with missing data on age, 12,187 participants remained. As various clinical conditions are known to alter gut microbiome composition, analyses were restricted to healthy individuals, defined as participants with no history of inflammatory bowel disease (IBD) or diabetes and no antibiotic use within the past year, yielding a subcohort of 7,214 participants. Since participants were drawn from multiple countries, analyses were further restricted to participants residing in the United States to mitigate the confounding influence of geographical variation on microbiome composition (Yatsunenko et al., 2012), resulting in a sample of 4,817 individuals. Finally, as our primary hypothesis concerns microbial dynamics in adulthood, only participants aged 18 years or older were retained, yielding a final analytic sample of 4,496 participants.

To investigate the dynamics of the gut microbiome across the adult lifespan, participants were stratified into seven age groups: 18–30, 31–37, 38–44, 45–51, 52–57, 58–64, and 65+ years, with sample sizes of 609, 640, 593, 677, 627, 705, 645, respectively. The upper age boundary of the oldest group (65+) was defined in accordance with existing literature (Biagi et al., 2010). The remaining breakpoints were determined empirically based on the age distribution of the sample, such that all groups had similar sample sizes. This balanced grouping strategy was employed to ensure that statistical comparisons across age groups reflect true biological differences in microbiome composition rather than artifacts arising from substantially different sample sizes.

**The AGP microbiome data processing**. After fecal samples were self-collected by participants, DNA was extracted using the MoBio PowerSoil kit (now Qiagen). The V4 hypervariable region of the 16S rRNA gene was amplified with a unique barcode attached to each primer to enable multiplexed sequencing. Amplicons were sequenced on the Illumina HiSeq/MiSeq platform, generating single-end 150 bp reads. Raw sequencing reads were demultiplexed by barcode and subjected to quality filtering in QIIME 2 (Caporaso et al., 2010), including truncation of reads at positions with Phred quality scores below 20, removal of reads falling below a minimum length threshold, and discarding of reads exceeding a maximum expected error rate. Denoising and amplicon sequence variant (ASV) inference were performed using Deblur (Amir et al., 2017), which corrects for sequencing errors at single-nucleotide resolution without clustering reads by similarity. Taxonomy was then assigned to each ASV using DADA2 (Callahan et al., 2016). The resulting feature table was further filtered to remove ASVs of mitochondrial or chloroplast origin, low-frequency features appearing in fewer than a minimum number of samples, and samples with a sequencing depth below 1,000 reads. Finally, raw read counts were converted to relative abundances by dividing each ASV count by the total read count for the corresponding sample, yielding a compositional relative abundance OTU table in which all values within a sample sum to unity.

**Statistical comparisons**. We used the James’s test (Tsagris et al., 2017) to compare the mean of the relative abundance across age groups (**Figure 2C**). To perform rCCLasso on the AGP data, we added a pseudo-abundance of 10^−7^ to zeros as the logarithm of 0 is not meaningful. This value was chosen because the smallest nonzero relative abundance across age groups was on the order of 10^−6^. To assess the sensitivity of our results to this choice, we conducted an additional analysis using a half-minimum pseudo-count, a data-adaptive strategy that has been used in microbiome applications (Boulund et al., 2026). Specifically, for each age group, we set the pseudo-count to one-half of the minimum nonzero relative abundance observed within that group. As summarized in **Table 1**, both the estimated correlation matrices and the corresponding −log_10_(*P*) values remained highly consistent across the two pseudo-count strategies, suggesting that the primary conclusions of rCCLasso are robust to the choice of pseudo-count. Detailed scatterplots comparing the two strategies are provided in **Supplementary Section S4** (**Supplementary Figure 3**). Given the relatively small number of nodes (13 classes), we used the Wilcoxon signed-rank test (paired-group test) to compare the nodes’ degree between age groups (**Figure 3A**). A significance level of *α* = 0.05 was considered. All statistical analyses were conducted using R version 4.4.0.

**Table 1:** Sensitivity analysis of pseudo-count selection across age groups.

Age group	Sample size *n*	Corr. of $\hat{R}$	Corr. of −log_10_(*P*)
18–30	609	0.955	0.940
31–37	640	0.969	0.871
38–44	593	0.979	0.957
45–51	677	0.976	0.967
52–57	627	0.980	0.917
58–64	705	0.963	0.938
65+	645	0.973	0.947

Similarity between the two pseudo-count strategies was evaluated using the Pearson correlation of the estimated correlation coefficients and the corresponding −log_10_(*P*) values.

## 4 Discussion

In this study, we developed rCCLasso, a robust correlation-based framework for microbial network analysis that addresses the numerical instability of the widely used CCLasso method. By aggregating results from multiple runs with different random seeds, rCCLasso stabilizes correlation estimates and associated statistical inference, leading to more reproducible and interpretable microbial interaction networks from compositional data. Through simulation studies, we demonstrate that rCCLasso improves type-I error control while maintaining comparable or improved power relative to CCLasso. Applying rCCLasso to the American Gut Project cohort of over 4,000 individuals, we identified several previously overlooked age-related changes in microbial network structure. These findings highlight the value of examining microbial interaction dynamics, beyond taxonomic abundance and diversity, in advancing our understanding of how the gut microbiome evolves across the human lifespan.

Our analysis is subject to several limitations of the American Gut Project (AGP) cohort. Although the AGP represents one of the largest publicly available microbiome datasets spanning a broad adult age range, its sequencing depth is relatively modest compared to more recent microbiome studies. As a result, the data exhibit substantial sparsity, with a high proportion of zero counts (approximately 99% at the genus level). Such excess zeros pose challenges for reliable correlation estimation, particularly at finer taxonomic resolutions where many taxa are observed infrequently across individuals. To mitigate instability arising from sparsity, we conducted our primary network analyses at the class level rather than the genus level. While this aggregation improves statistical stability, it may obscure more granular interaction patterns that occur at lower taxonomic levels. In addition, fecal samples in the AGP were self-collected by participants. Although standardized collection protocols and careful processing procedures were implemented, some degree of sample contamination or variability in collection conditions is difficult to avoid in large-scale citizen-science studies. Such variability may introduce additional noise into the data and potentially influence the inferred microbial associations.

Another important consideration is the reproducibility of microbiome–aging network patterns across independent cohorts. Ideally, findings from the present study would be validated in other large-scale microbiome datasets spanning a broad adult age range. However, such single-cohort studies remain relatively rare. Many existing investigations of the gut microbiome–aging relationship achieve large sample sizes by pooling data from multiple studies (Chen et al., 2022; Galkin et al., 2020). While this strategy increases statistical power, it can introduce substantial batch effects arising from differences in study design, sequencing protocols, and data processing pipelines, which may complicate cross-study comparisons and interpretation. As larger and more deeply sequenced microbiome cohorts covering wide age ranges become available, applying rCCLasso to these datasets will provide valuable opportunities to assess the generalizability of the observed network patterns and to further investigate microbial interaction dynamics at finer taxonomic resolution.

A related consideration concerns how age-related microbiome dynamics are represented. In this study, we evaluated microbial network changes using pre-defined age groups, dividing participants into seven categories with approximately balanced sample sizes. This stratification provides a practical and interpretable framework for comparing microbial interaction patterns across different stages of adulthood while maintaining sufficient statistical stability for network estimation. At the same time, aging is inherently a continuous biological process, and grouping may smooth over more gradual transitions in microbial interactions. Future methodological developments could extend this framework by modeling microbial correlation dynamics as continuous functions of age. Such approaches would require time-varying or age-dependent correlation models that accommodate both compositional constraints and high dimensionality. Flexible methods, such as spline-based or kernel-based approaches, may offer promising directions for capturing gradual changes in microbial connectivity across adulthood.

Another methodological limitation concerns the treatment of potential confounders. In the present analysis, we addressed major confounding factors at the data preprocessing stage by restricting the cohort to participants residing in the United States and excluding individuals with self-reported clinical conditions or recent antibiotic use. While these restrictions help reduce heterogeneity, they cannot fully account for other sources of variation such as diet, lifestyle, medication history, or sequencing-related technical factors. Ideally, future methodological developments should incorporate confounder adjustment directly into the network estimation framework, allowing microbial associations to be inferred conditional on relevant covariates. Integrating compositional network estimation with regression-based adjustment or conditional correlation modeling may therefore provide a more principled strategy for disentangling microbial interactions from confounding influences.

## Data Availability

The de-identified AGP datasets analyzed in this study can be found in the Github repository “American-Gut”: https://github.com/biocore/American-Gut/tree/master.
