# Supplementary material for: rCCLasso: a robust framework for microbial correlation network analysis reveals age-related microbial dynamics
Source: Front Cell Infect Microbiol. 2026 Aug 17;16:1828471. doi: 10.3389/fcimb.2026.1828471 (PMC13526604; doi:10.3389/fcimb.2026.1828471)
Supplement: Supplementary file 1 [file DataSheet1.pdf]

Supplementary Material for rCCLasso: A Robust Framework for Microbial Correlation Network Analysis Reveals Age-Related Microbial Dynamics

Tianyi Xie[1], Jie Zhou[2], and Yue Wang[3,*]

[1]Department of Statistics, Oregon State University, Corvallis, OR, USA
[2]School of Mathematical Sciences, Capital Normal University, Beijing, PR China
[3]Department of Biostatistics and Informatics, University of Colorado Anschutz Medical Campus, Aurora, CO, USA

[*]Correspondence: Yue Wang, `yue.2.wang@cuanschutz.edu`

This Supplementary Material provides additional methodological details, simulation results, and sensitivity analyses supporting the main text. Specifically, Section S1 presents the bootstrap de-biased inference procedure implemented in CCLasso. Sections S2 and S3 report additional simulation studies under the logistic-normal–multinomial model and under different values of the repeated-run parameter B, respectively. Section S4 provides a sensitivity analysis for pseudo-count selection; and Section S5 displays the remaining age-group-specific microbial correlation networks not shown in the main text.

S1 Bootstrap de-biased inference procedure in CCLasso

Algorithm 1 Bootstrap De-biased Inference Procedure in CCLasso

Require: Compositional data matrix $\boldsymbol{X} \in \mathbb{R}^{n \times p}$; number of bootstrap samples B; nominal significance level α

Ensure: Debiased correlation matrix $\widehat{\boldsymbol{R}}^{de}$; p-value matrix $\widehat{\boldsymbol{P}}^{de}$

Initialize storage for bootstrap correlation estimates $\{\widehat{\boldsymbol{R}}^{(b)}\}_{b=1}^{B}$ and $\{\widehat{\boldsymbol{v}}^{(b)} = \mathrm{diag}(\widehat{\boldsymbol{\Sigma}}^{(b)})\}_{b=1}^{B}$.

for $b = 1$ to B **do**

  Generate a bootstrap sample $\boldsymbol{X}^{(b)}$ from $\boldsymbol{X}$

  Apply CCLasso to $\boldsymbol{X}^{(b)}$ and obtain correlation estimate $\widehat{\boldsymbol{R}}^{(b)}$ and variance vector $\widehat{\boldsymbol{v}}^{(b)}$

end for

Compute the bootstrap-averaged variance estimator by averaging the variance vectors across bootstrap replicates:

$$\widehat{\boldsymbol{v}}^{\mathrm{boot}} = \frac{1}{B} \sum_{b=1}^{B} \widehat{\boldsymbol{v}}^{(b)}.$$

Simulate the artificial null log-abundance vector $\log \boldsymbol{Y}^{\mathrm{art}} \sim N\big(\mathbf{0}, \mathrm{diag}(\widehat{\boldsymbol{v}}^{\mathrm{boot}})\big)$, and obtain the corresponding relative abundance $\boldsymbol{X}^{\mathrm{art}}$ via the softmax transformation.

Apply the standard correlation estimator to $\log \boldsymbol{Y}^{\mathrm{art}}$ to obtain the reference correlation estimate $\widehat{\boldsymbol{R}}^{\mathrm{art0}} = \mathrm{cor}\big(\log \boldsymbol{Y}^{\mathrm{art}}\big)$, and apply CCLasso to $\boldsymbol{X}^{\mathrm{art}}$ to obtain the compositional correlation estimate $\widehat{\boldsymbol{R}}^{\mathrm{art}}$.

Estimate the global shrinkage bias via Fisher's z-transformation

$$\widehat{b} = \mathrm{median} \left| \log \frac{1 + \widehat{R}_{ij}^{\mathrm{art0}}}{1 - \widehat{R}_{ij}^{\mathrm{art0}}} - \log \frac{1 + \widehat{R}_{ij}^{\mathrm{art}}}{1 - \widehat{R}_{ij}^{\mathrm{art}}} \right|,$$

over all eligible entries.

Construct the final debiased correlation matrix $\widehat{\boldsymbol{R}}^{de}$ by correcting and averaging the initial bootstrap estimate $\widehat{\boldsymbol{R}}^{(b)}$ with the estimated compositional bias.

Construct the debiased correlation matrix by

$$\widehat{R}_{ij}^{de} = \frac{1}{B} \sum_{b=1}^{B} \tanh \left(\frac{\log \frac{1 + \widehat{R}_{ij}^{(b)}}{1 - \widehat{R}_{ij}^{(b)}} + \mathrm{sign}\Big(\widehat{R}_{ij}^{(b)}\Big)\, \widehat{b}}{2} \right).$$

Compute the final p-value matrix $\widehat{\boldsymbol{P}}^{de} = (\widehat{p}_{ij}^{de})$ by evaluating the t-statistics derived from $\widehat{\boldsymbol{R}}^{de}$ against the Student's t-distribution with $n-2$ degrees of freedom

return $\widehat{\boldsymbol{R}}^{de}$, and $\widehat{\boldsymbol{P}}^{de}$

S2 Additional Simulation Results under the LNM Model

To further evaluate the robustness of the proposed method under a more biologically realistic data-generating mechanism, we additionally conducted simulation studies using a logistic-normal–multinomial (LNM) model (Xia et al., 2013; Tian et al., 2023). Specifically, for each subject $i = 1, \ldots, n$, a latent log-abundance vector was first generated according to

$$Z_i \sim N(\mu, \Sigma),$$

where Σ denotes the prespecified correlation structure. The latent Gaussian vector was then transformed to the underlying relative abundance vector

$$\boldsymbol{q}_i = (q_{i1}, \ldots, q_{ip})^\top, \qquad q_{ij} = \frac{\exp(Z_{ij})}{\sum_{k=1}^{p} \exp(Z_{ik})}, \quad j = 1, \ldots, p,$$

which served as the multinomial sampling probabilities. Finally, the observed count vector was generated from

$$X_i \sim \text{Multinomial}(M_i, q_i),$$

where M_i denotes the sequencing depth (library size) for subject i. Compared with the logistic-normal (LN) model used in the main text, the LNM model explicitly accounts for library size variation and therefore more closely mimics the microbiome sequencing process. In the LNM simulations, the sequencing depth M_i was sampled uniformly from the integers between $100p$ and $200p$. Unless otherwise specified, all remaining simulation settings were identical to those in the LN simulations. Figures S1 present the empirical Type I error and statistical power under two data dimensions, $(n, p) = (500, 20)$ and $(n, p) = (500, 50)$, respectively. Overall, the LNM simulations lead to conclusions highly consistent with those obtained under the LN model, demonstrating that the proposed rCCLasso framework is robust across different data-generating mechanisms.

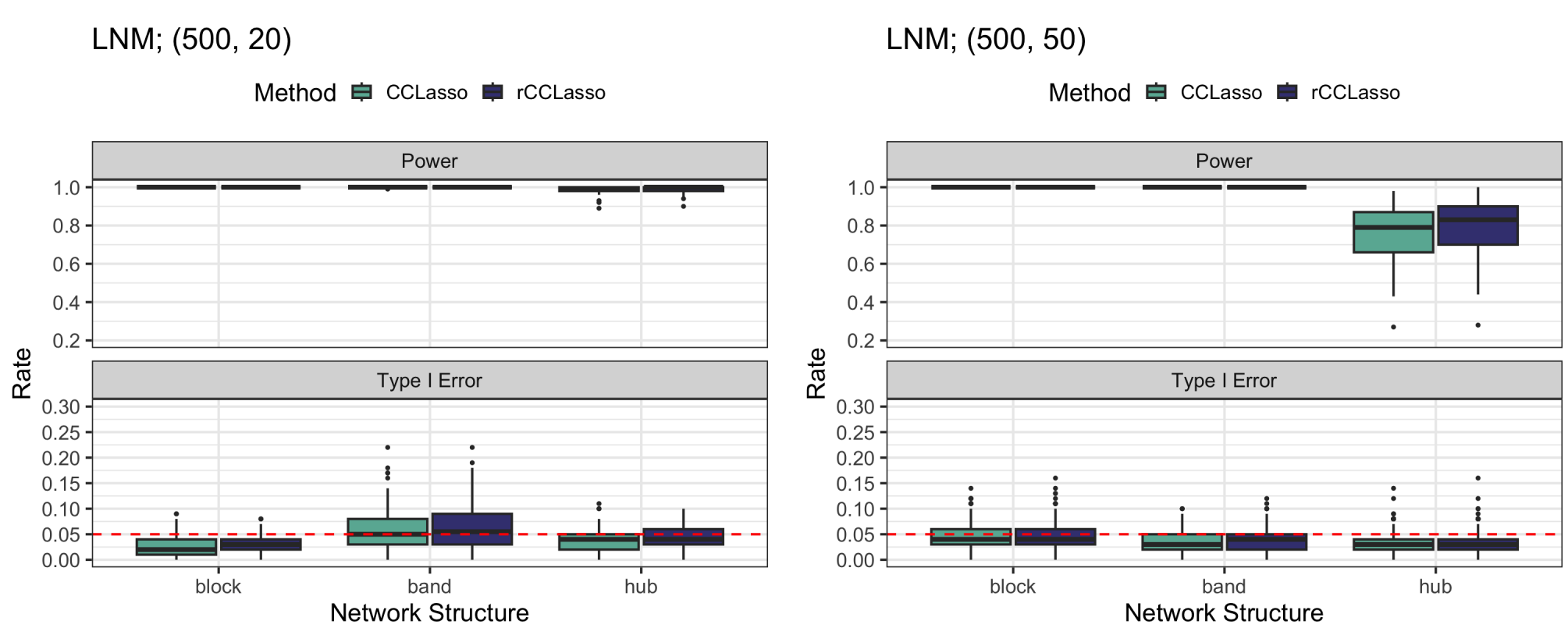

Figure S1: Simulation results under the logistic-normal–multinomial (LNM) model. Comparison of CCLasso and rCCLasso under two simulation settings: $(n, p) = (500, 20)$ (left) and $(n, p) = (500, 50)$ (right). Three correlation structures (block, band, and hub) were considered. The upper panels show the entry-wise empirical power and the lower panels show the entry-wise empirical Type I error.

S3 Additional Simulation Results for Different Numbers of Repeated Runs

The number of repeated runs, B, is the primary tuning parameter of rCCLasso. Throughout the main text, we adopted $B = 100$ as the default setting to balance computational efficiency and estimation stability. To assess the sensitivity of the proposed method to the choice of B, we additionally considered $B = 50$ and $B = 200$, while keeping all other simulation settings identical to those in the main text. As shown in Figure S2, the simulation results are highly consistent across all three choices of B, with similar empirical Type I error rates and statistical power under both simulation settings and all correlation structures. These results demonstrate that the proposed rCCLasso framework is robust to the choice of B.

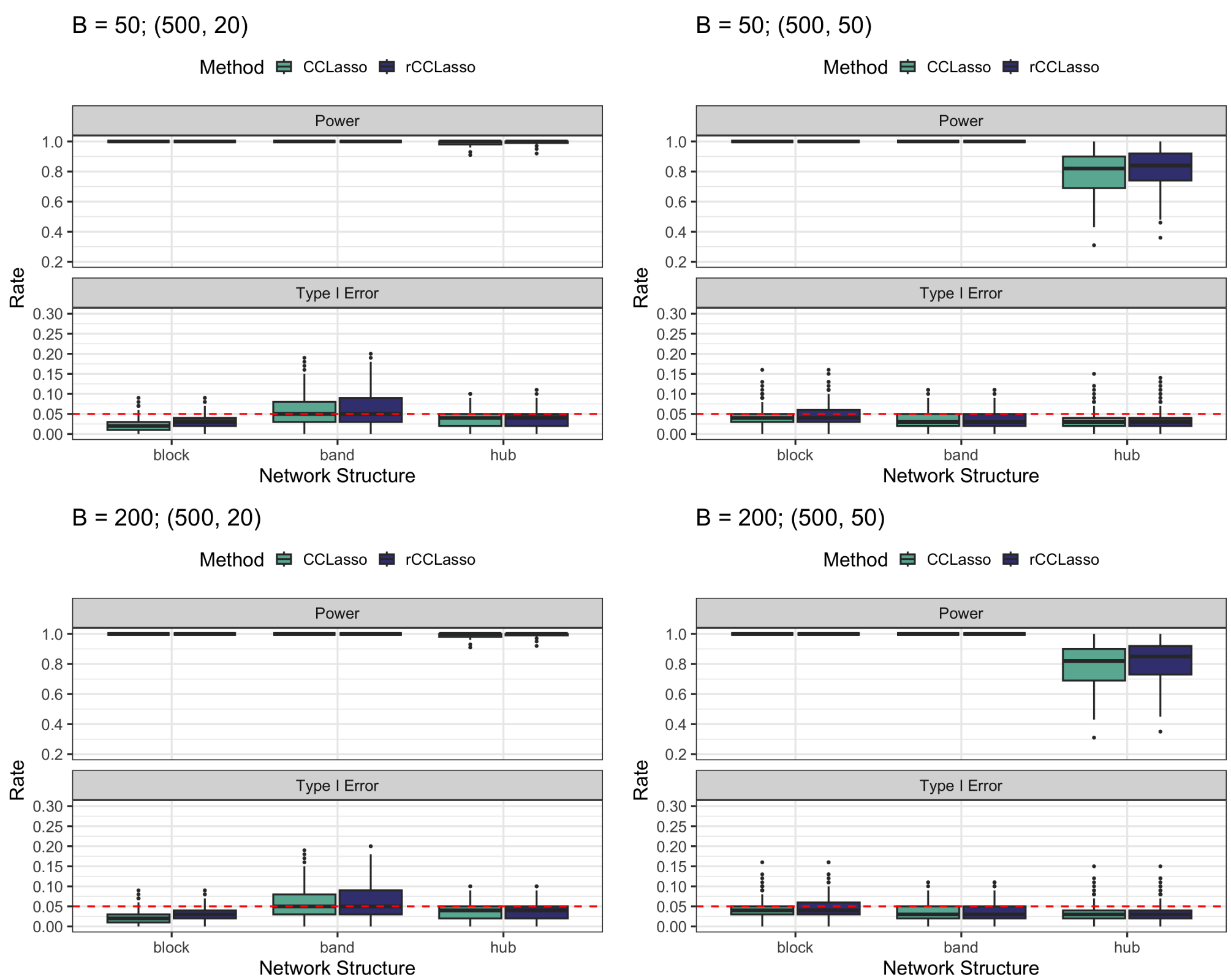

Figure S2: Sensitivity analysis with respect to the number of repeated runs (B). Entry-wise empirical power and Type I error of CCLasso and rCCLasso under two simulation settings with $B = 50$ (top) and $B = 200$ (bottom). The results are highly consistent with those obtained using the default setting $B = 100$.

S4 Pseudo-count Sensitivity Analysis

Similar to the original CCLasso framework, we assume that zero entries in the observed OTU table do not necessarily represent true biological absence but may instead result from limited sequencing depth or measurement resolution. For count data, it is common practice to add a small pseudo-count (e.g., 0.5 or 1) before applying log-ratio transformations. Accordingly, the original CCLasso implementation adds a pseudo-count of 0.5 prior to normalization. In our study, the real-data analysis was conducted using the publicly available AGP dataset, for which only relative abundance (compositional) data are available and the original sequencing counts are not provided. Consequently, the pseudo-count strategy used for count data (e.g., adding 0.5 or 1) is not applicable. Across all seven age groups, the smallest nonzero class-level relative abundance was on the order of 10^{-6}. We therefore added a pseudo-count of 10^{-7}, one order of magnitude smaller than the smallest observed nonzero abundance, which minimally perturbs the original compositions while ensuring that logarithmic transformations are well defined.

To assess the sensitivity of our results to this choice, we conducted an additional analysis using a half-minimum pseudo-count, a data-adaptive strategy that has been used in microbiome applications (Boulund et al., 2025). Specifically, for each age group, we set the pseudo-count to one-half of the minimum nonzero relative abundance observed within that group. This approach scales the offset according to the data and therefore provides a useful sensitivity analysis.

We re-ran the entire rCCLasso pipeline under this alternative pseudo-count specification and compared the resulting correlation networks with those obtained using the fixed 10^{-7} offset. Ss shown in Figure S3, the estimated correlation matrices remained highly consistent across the two pseudo-count strategies, with Pearson correlations ranging from 0.955 to 0.980. The corresponding edge-wise statistical evidence was similarly preserved, with Pearson correlations between the $-\log_{10}(P)$ values ranging from 0.871 to 0.967. The slightly lower correlations for the transformed P-values were primarily driven by a small number of edges with extremely small P-values, for which minor numerical differences are amplified on the $-\log_{10}$ scale. Collectively, these results suggest that the primary conclusions of rCCLasso are robust to the choice of pseudo-count.

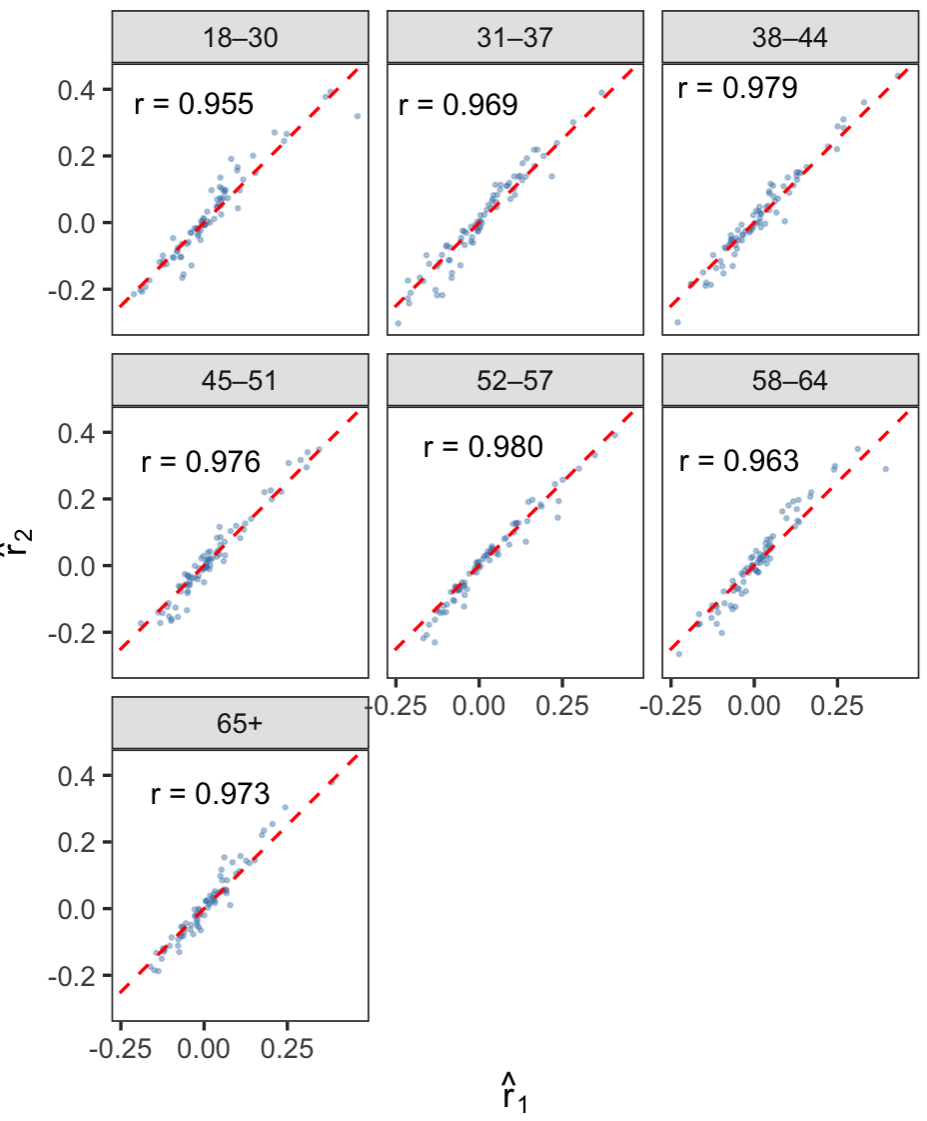

(a) Estimated correlation coefficients

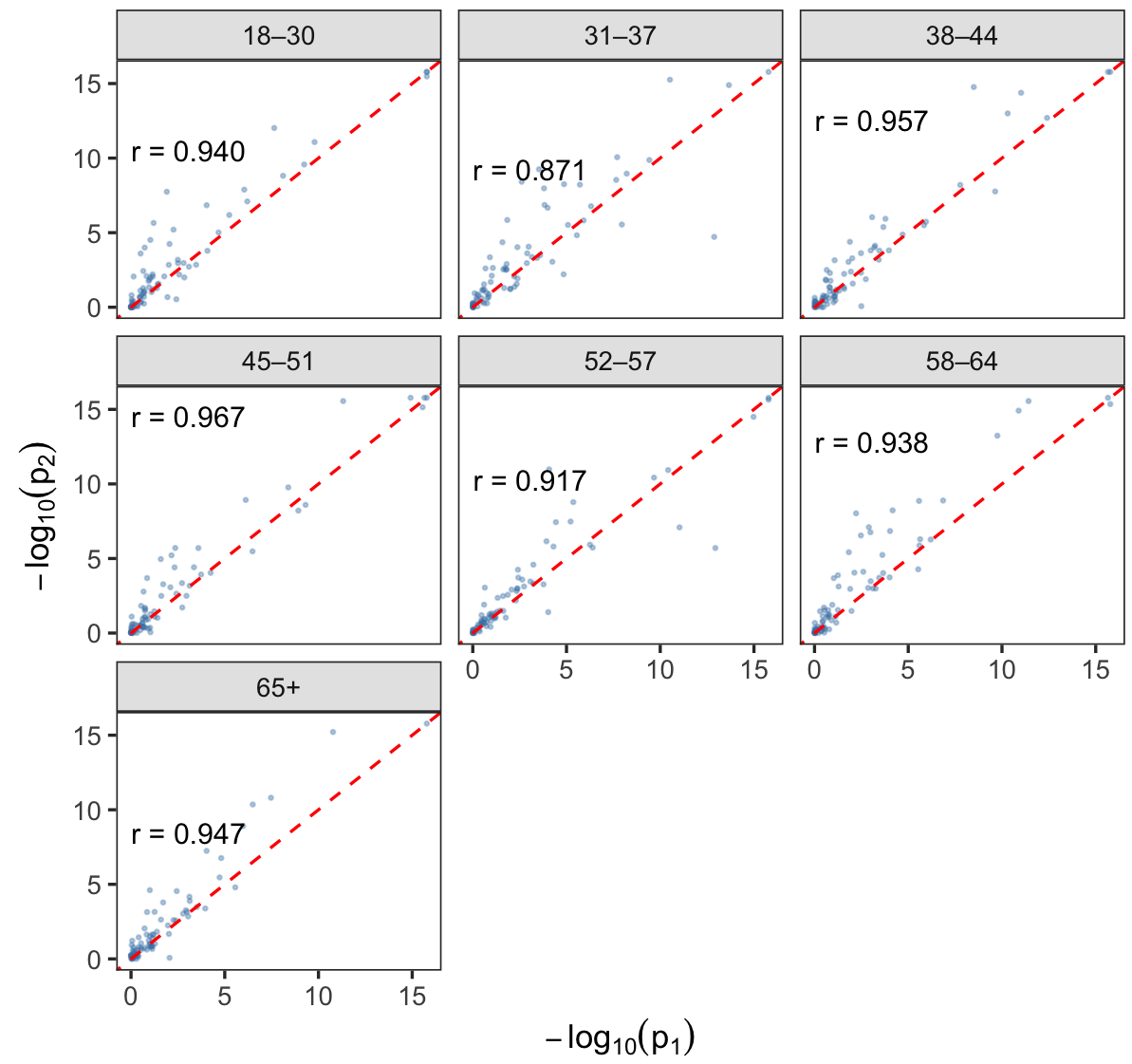

(b) Edge-wise $-\log_{10}(P)$ values

Figure S3: Sensitivity analysis of pseudo-count selection across age groups. Comparison of (a) the estimated correlation coefficients and (b) the corresponding edge-wise $-\log_{10}(P)$ values obtained using two pseudo-count strategies: a fixed offset of 10^{-7} and one-half of the minimum nonzero relative abundance within each age group. The red dashed line denotes the identity line ($y = x$), and the Pearson correlation coefficient (r) is reported for each age group.

S5 Additional Age-Group-Specific Networks

For visualization purposes, only the 18–30 and 65+ age-group-specific microbial correlation networks are shown in the main text. The remaining age-group-specific networks are provided in Figure S4 using the same visualization settings.

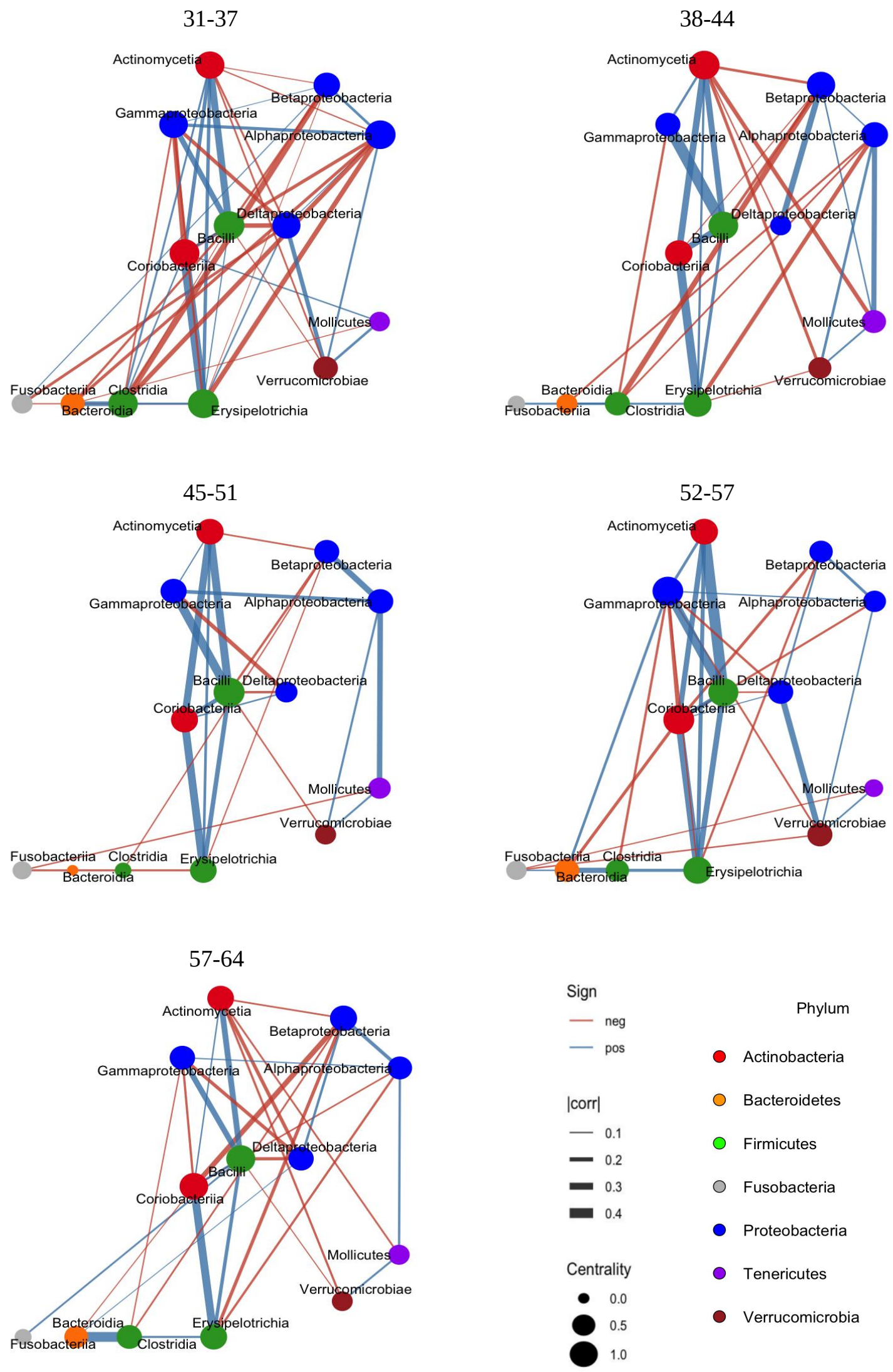

Figure S4: Additional age-group-specific microbial correlation networks inferred by rCCLasso for the 31–37, 38–44, 45–51, 52–57, and 58–64 age groups. Network visualization follows the same convention as Figure 3C: nodes are colored by phylum and sized according to node degree; red and blue edges represent positive and negative correlations, respectively, and edge width is proportional to the absolute correlation strength.

References

Boulund, U., Thorsen, J., Larsen, F., Poulsen, C. S., Widdowson, M., Jiang, J., et al. (2025). Infant gut anellovirus composition associates with early life factors and childhood atopic disease. *Nature Communications*

Tian, C., Jiang, D., Hammer, A., Sharpton, T., and Jiang, Y. (2023). Compositional graphical lasso resolves the impact of parasitic infection on gut microbial interaction networks in a zebrafish model. *Journal of the American Statistical Association* 118, 1500–1514

Xia, F., Chen, J., Fung, W. K., and Li, H. (2013). A logistic normal multinomial regression model for microbiome compositional data analysis. *Biometrics* 69, 1053–1063
